# Supplementary material for: SntB Affects Growth to Regulate Infecting Potential in Penicillium italicum
Source: J Fungi (Basel). 2024 May 21;10(6):368. doi: 10.3390/jof10060368 (PMC11204802; doi:10.3390/jof10060368)
Supplement: Supplementary file 1 [file jof-10-00368-s001.zip › jof-2999940-supplementary.pdf]

Journal of Fungi

MDPI

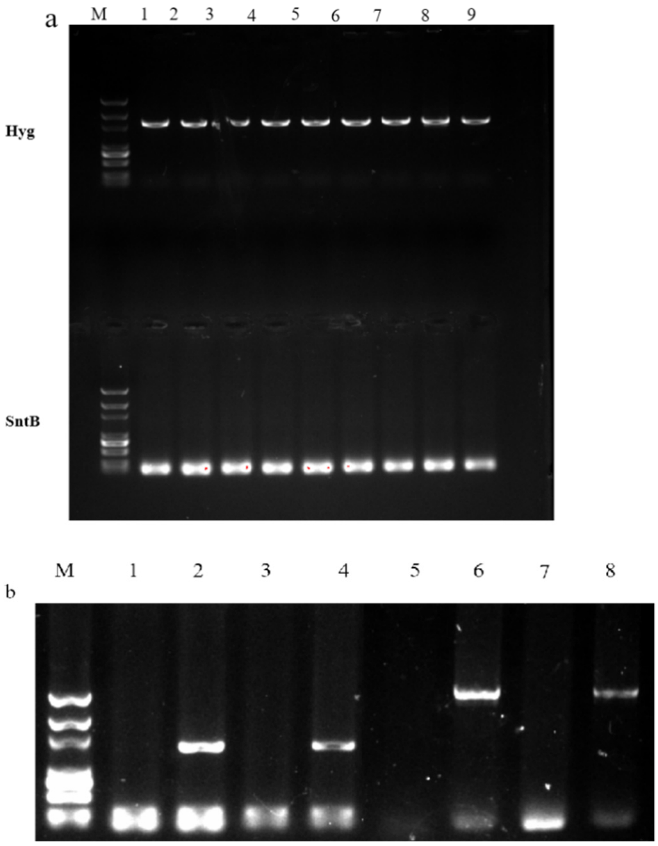

Figure S1. Nucleic acid electrophoretic graph of *SntB* knock out strain. **Note**: a: Preliminary validation of resistance genes and target genes in transformants; M:5000bp marker. b: 1,3: Validation of the target gene for *ΔPiSntB*.2;4: Validation of the *ΔPiSntB* resistance gene.5,7: Validation of *ΔPiSntB* using the upper homologous arm+target gene and the lower homologous arm+target gene, respectively.6;8: Controls for the upper and lower homologous arms+target gene, respectively. M:5000bp marker.

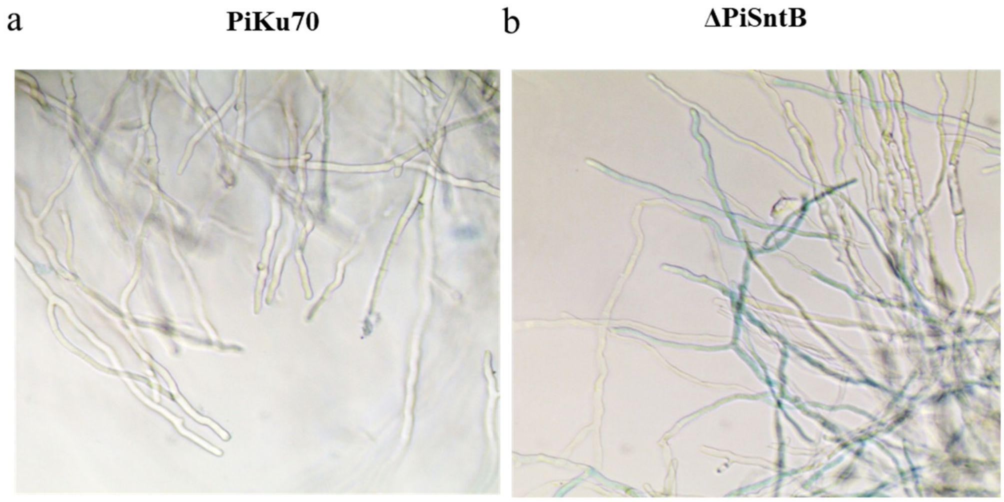

Figure S2. Evans blue staining situation. **Note**: a-b: Evans blue stained mycelia.

Table S1. Construction of knockout vectors and primers for transformant validation.

Primers	Sequence（5′ to 3′）	Explanation
SntB-Up-F	catgattacgaattcgagctcCCAGTTGGATTCCCGA-GATT	For amplification of the 5' homology arm of *SntB*, 1383bp, lowercase letters are sequences homologous to vector and resistance fragments, bolded parts are SacI digestion sites
SntB-Up-R	caatatcatcttctgtcgacACCTATGTCCTTTCCTATTT-GCTT	
Hyg-F	GTCGACAGAAGATGATATTG	For amplification of hygromycin B resistance fragments
Hyg-R	CTAGAAAGAAGGATTACCTC	
SntB-Down-F	gaggtaatccttctttctagAAGAGGACACGCTCAATGG-TAG	Used to amplify the 3' homology arm of *SntB*, 1549bp, lowercase letters are sequences homologous to vector and resistance fragments, bolded parts are XbaI digestion sites
SntB-Down-R	tgcctgcaggtcgactctagaATTTTGTGGAGATAACAC-GGAA	
SntB-F	atgaccactcccaactctcc	The sequence of *SntB* totaled 4944bp
SntB-R	ttatgagagaaggttcttcagcg	

Table S2. The primers of RNA-Seq genes.

Primers	Sequence（5′ to 3′）	Explanation
Actin-F	GAAGGAGCAAGAGCAGTG	Housekeeping gene
Actin-R	GATGTTGATGTTCGTAAGGA	
PITC_045240-F	TTCGTGTAACCTTGTCTCTG	Glucose-repressible protein Grg1
PITC_045240-R	GTCCTTCTTGTCGACAACGG	
PITC_030020-F	GTATACCGCCGACTGGCAA	Oxoglutarate/iron-dependent dioxygenase
PITC_030020-R	CTTCACCGGTGGGACTACC	
PITC_038780-F	GGCAAAGATGATGACTGGTA	Short-chain dehydrogenase/reductase SDR
PITC_038780-R	CGTGTCATGGAAATAACACC	
PITC_071360-F	ACAGTGACGACTTCCTGCAG	Actin-binding, cofilin/tropomyosin type
PITC_071360-R	GGCGTCTTCGGTAACCGA	
PITC_081320-F	CCAAAAGAAAGCTTACTCCC	NADH-ubiquinone oxidoreductase
PITC_081320-R	GCGGAAAATAATGCCCAGC	
PITC_047390-F	GGGCGAGGATTCATGGGATA	Gene homologous to *LaeA*
PITC_047390-R	GTCGAGTACCAGCTAGCCAT	
PITC_003060-F	TCGCGCATCCATAACAATCC	Gene homologous to *CreA*
PITC_003060-R	TGGAGAAGGAGTGAGGAGGA	
PITC_013310-F	GCACTCCTACGGCAATGTTT	Gene homologous to *PacC*
PITC_013310-R	TTGAGGAAGGGAAGCTGGAG	

Table S3. The differentially expressed genes (DEGs) after *SntB* knockout in *P. italicum*.

Gene name	Gene description	Log_2FC	Padjust	FPKM	
				ΔPiSntB	PiKu70
	Cellular structure and material transport				
PITC_058840	Major facilitator superfamily domain, general substrate transporter	4.17	1.02E-123	160.09	8.30
PITC_005580	Major facilitator superfamily domain, general substrate transporter	2.73	1.18E-58	652.73	91.76
PITC_057950	Dynamin	4.97	8.67E-143	104.27	3.09
PITC_083340	Dynamin	5.03	1.96E-53	29.33	0.84
PITC_083180	Amino acid transporter, transmembrane	-1.06	3.59E-26	526.56	1023.70

PITC_006380	C4-dicarboxylate transporter/malic acid transport protein	-1.88	8.01E-05	105.50	362.79
PITC_040980	Vacuolar protein sorting-associated protein 62	-1.02	5.36E-08	89.90	169.45
PITC_033280	Permease, cytosine/purine, uracil, thiamine, allantoin	-2.24	2.46E-62	106.08	466.04
PITC_066290	Sodium/calcium exchanger membrane region	-1.35	1.29E-31	53.64	126.59
PITC_008500	Cell wall beta-glucan synthesis	3.26	1.47E-36	146.09	14.40
PITC_071360	Actin-binding, cofilin/tropomyosin type	-1.34	1.57E-03	767.87	1945.20
PITC_003900	Tubulin	-0.62	8.91E-13	1286.61	1848.23
PITC_002540	Concanavalin A-like lectin/glucanases superfamily	2.29	4.02E-54	211.90	40.77
PITC_041170	Concanavalin A-like lectin/glucanases superfamily	1.41	5.94E-15	535.06	189.68
PITC_015600	Hydrophobin	1.23	1.44E-02	458.47	188.56
PITC_001010	Hydrophobin	2.38	3.40E-10	198.12	39.10
PITC_077120	Hydrophobin	2.07	2.12E-04	301.46	69.71
	Lipid metabolism				
PITC_032560	Alpha/beta hydrolase fold-3	2.69	6.55E-44	163.31	23.79
PITC_051810	Lipase, class 3	8.41	3.13E-11	31.34	0.07
PITC_083320	Lipase, secreted	1.60	1.82E-31	104.39	32.32
PITC_038780	Short-chain dehydrogenase/reductase SDR	4.06	1.42E-97	501.14	28.41
PITC_052730	Short-chain dehydrogenase/reductase SDR	2.11	1.66E-08	1179.88	258.42
PITC_020530	Acyl-CoA N-acyltransferase	-0.67	1.42E-07	107.35	159.57
PITC_098660	Fatty acid desaturase, type 1, core	-2.34	4.46E-34	47.43	223.11
PITC_031890	Fatty acid hydroxylase	-1.39	6.39E-17	98.21	240.92
PITC_023140	Acyl transferase/acyl hydrolase/lysophospholipase	-2.60	7.18E-68	11.24	63.13
PITC_090390	Acyl transferase/acyl hydrolase/lysophospholipase	-1.29	3.75E-32	66.69	150.44
PITC_013770	Phospholipid methyltransferase	-0.66	4.25E-05	136.35	206.36
	Carbohydrate metabolism				
PITC_008490	Alpha-amylase, fungi	2.17	1.49E-32	120.67	24.92
PITC_069100	Carbon-nitrogen hydrolase	1.07	2.91E-10	225.71	100.59
PITC_029870	Carbon-nitrogen hydrolase	1.45	9.72E-18	4563.27	1570.54
PITC_058850	Glycoside hydrolase family 3	2.95	7.76E-106	253.59	30.60
PITC_003820	Glycoside hydrolase, family 12	2.01	2.55E-22	157.04	36.91
PITC_055440	Glucose/ribitol dehydrogenase	3.62	2.93E-103	248.76	19.10
PITC_045240	Glucose-repressible protein Grg1	5.64	7.99E-05	301.46	5.96
PITC_061580	Glucose-repressible protein Grg1	2.60	9.12E-10	1137.65	215.37
PITC_095870	Phosphoenolpyruvate carboxykinase, ATP-utilizing	1.72	1.74E-62	349.58	98.74
PITC_097960	6-phosphofructokinase, eukaryotic type	-1.61	2.34E-29	316.69	898.17
PITC_089720	Enolase, C-terminal	-1.10	1.23E-20	4222.37	8465.97
PITC_061910	Thiamine pyrophosphate enzyme, C-terminal TPP-binding	-1.71	4.67E-30	3563.47	10884.34
PITC_041290	Phosphoglycerate kinase	-1.14	3.52E-16	1723.85	3555.56
	Amino acid /protein metabolism				
PITC_001990	Amidase	2.00	2.22E-28	25.92	6.01

PITC_079890	Amidase	1.50	4.51E-43	363.41	119.92
PITC_029880	D-amino-acid oxidase	1.18	2.09E-25	2292.59	948.66
PITC_072940	Copper amine oxidase	2.46	1.80E-80	117.73	19.92
PITC_025260	Copper amine oxidase, N2-terminal	1.67	1.91E-28	1256.88	368.38
PITC_039780	Serine hydrolase FSH	4.65	1.71E-121	555.16	21.35
PITC_008170	Serine hydrolase FSH	2.50	1.52E-25	49.83	8.28
PITC_025820	Peptidase S10, serine carboxypeptidase	5.29	1.05E-299	360.64	8.54
PITC_045460	Peptidase S8/S53, subtilisin/kexin/sedolisin	4.95	2.93E-135	139.51	4.20
PITC_073660	Ribosomal protein S23/S29, mitochondrial	-0.28	2.28E-02	137.11	155.39
PITC_089630	Ribosomal protein S36, mitochondrial	-0.44	2.18E-02	254.33	350.53
	Oxidative stress and immune response				
PITC_093440	DJ-1 domain, InhA-type	4.89	3.05E-30	438.44	14.11
PITC_012420	Glutathione S-transferase, N-terminal	1.17	3.82E-14	256.40	108.56
PITC_033490	Glutathione S-transferase, N-terminal	2.88	3.73E-83	380.07	49.43
PITC_031710	Glutathione S-transferase/chloride channel, C-terminal	1.89	4.10E-34	222.74	56.92
PITC_003880	Glutathione S-transferase/chloride channel, C-terminal	1.63	8.35E-46	1576.81	487.14
PITC_037590	Serine-threonine protein kinase 19	0.29	2.95E-02	158.67	121.96
PITC_052690	Cytochrome P450	8.85	2.43E-15	38.88	0.08
PITC_005950	Cytochrome P450, E-class, CYP52	1.73	2.89E-29	375.36	105.28
PITC_027470	Aldehyde dehydrogenase, N-terminal	1.65	5.61E-57	577.61	171.95
PITC_032430	Aldehyde/histidinol dehydrogenase	1.59	1.84E-61	2003.95	621.14
PITC_000490	Oxoglutarate/iron-dependent dioxygenase	5.66	6.19E-64	103.31	1.93
PITC_024150	Oxoglutarate/iron-dependent dioxygenase	2.93	4.35E-09	278.24	34.15
PITC_030020	Oxoglutarate/iron-dependent dioxygenase	1.36	3.10E-16	1100.71	402.43
PITC_027250	Cupin 2, conserved barrel	4.85	2.85E-143	670.74	22.65
PITC_090090	HSP20-like chaperone	-2.65	4.24E-07	88.51	531.68
PITC_097450	Alcohol dehydrogenase, C-terminal	-1.50	1.66E-20	300.56	796.16
PITC_060320	Electron transfer flavoprotein-ubiquinone oxidoreductase	-0.50	2.07E-05	86.65	114.09
PITC_040670	Clp ATPase, C-terminal	-2.17	8.23E-10	98.87	411.85
PITC_000490	Oxoglutarate/iron-dependent dioxygenase	5.66	6.19E-64	103.31	1.93
	Energy metabolism				
PITC_030560	Mitochondrial inner membrane protein Mitofilin	-0.32	1.79E-03	72.90	84.35
PITC_066990	Mitochondrial outer membrane translocase complex, subunit Tom70	-0.45	2.41E-04	145.50	185.09
PITC_016450	Mitochondrial substrate/solute carrier	0.42	3.26E-03	312.67	220.82
PITC_088820	Mitochondrial carrier protein	1.13	1.39E-19	469.19	202.21
PITC_095900	Mitochondrial carrier protein	2.96	8.43E-24	34.98	4.23
PITC_081320	NADH-ubiquinone oxidoreductase, 20 Kd subunit	2.76	3.09E-94	636.58	89.49
PITC_023670	NADH:ubiquinone oxidoreductase, ASHI subunit	0.27	2.70E-02	729.99	596.24
PITC_014370	Phosphoesterase	1.23	6.31E-22	141.67	56.42
PITC_088100	Phosphoesterase	0.54	9.10E-07	1975.07	1267.41
PITC_058760	FAD-dependent pyridine nucleotide-disulfide oxidoreductase	3.89	8.75E-23	948.86	60.12

PITC_071230	Cytochrome c oxidase subunit VIIc	0.41	4.66E-03	12766.13	10222.97
PITC_075670	Cytochrome c, class IA/ IB	0.33	6.07E-03	2421.35	1903.81
PITC_070840	Cytochrome d ubiquinol oxidase, 14kDa subunit	0.30	2.06E-02	1901.58	1569.75
PITC_069900	Cytochrome b5	0.87	5.67E-14	1614.77	892.77
PITC_083400	Cytochrome b5	0.50	7.13E-05	1566.66	1102.41
PITC_056470	Cytochrome b-c1 complex subunit 8	0.33	1.70E-02	4232.03	3581.12
	Pathogenicity				
PITC_014830	Aegerolysin	-1.82	1.36E-04	947.56	3334.44
PITC_030230	Cutinase	-1.47	6.64E-36	771.99	2029.25
PITC_034160	Pectin lyase fold/virulence factor	-1.00	3.41E-16	1160.11	2175.50
